# Retrocuspid Papillae of the Gingiva: Clinical Nature and Controversies

**DOI:** 10.7759/cureus.106362

**Published:** 2026-04-03

**Authors:** N Megalaa, Thangadurai Maheswaran, Viswanathan Shanthi, V Sundar Raj, Karunamoorthy Vennila

**Affiliations:** 1 Pediatric and Preventive Dentistry, Mahatma Gandhi Postgraduate Institute of Dental Sciences, Puducherry, IND; 2 Oral and Maxillofacial Pathology, Adhiparasakthi Dental College and Hospital, Melmaruvathur, IND; 3 Oral Pathology and Microbiology, Tamil Nadu Government Dental College and Hospital, Chennai, IND; 4 Prosthodontics, Government Dental College, Pudukkottai, IND; 5 Periodontology, Vivekanandha Dental College for Women, Namakkal, IND

**Keywords:** epulis, giant cell fibroma, gingival lesions, lingual attached gingiva, multinucleated giant fibroblasts, oral developmental variants, oral pathology, retrocuspid papilla

## Abstract

The retrocuspid papilla (RCP) is an asymptomatic developmental soft-tissue nodule located in the attached gingiva, lingual to the mandibular cuspids. Frequently misinterpreted as a reactive or neoplastic gingival lesion, it remains under-recognized in dental practice despite its well-established benign nature. This editorial addresses its clinical presentation, epidemiology, histopathological characteristics, and core controversies surrounding its nosological classification and differential diagnosis from giant cell fibromas. Correct clinical identification eliminates the need for biopsy and prevents unnecessary interventions.

## Editorial

Introduction

The retrocuspid papilla (RCP) is a circumscribed sessile nodule that arises exclusively on the lingual attached gingiva adjacent to the mandibular cuspids, and its developmental origin is well established [1]. Despite a prevalence of 99% in certain pediatric cohorts, the RCP remains largely unfamiliar to dental practitioners, resulting in avoidable excisional biopsies [1,2]. It has been classified alongside Fordyce granules, geographic tongue, and lingual varices as an embryological variant of negligible clinical significance but of real diagnostic importance [3]. Its visual resemblance to fibrous reactive lesions and its histopathological overlap with giant cell fibroma (GCF) persistently generate controversy [4,5]. Nosological disagreement regarding whether the RCP represents a normal anatomical variant, developmental lesion, or merely a differential diagnosis entry further complicates clinical teaching and reporting [1,3]. The objective of this editorial is to offer a concise summary of the clinical nature and controversies surrounding the RCP to dental clinicians.

Clinical nature and epidemiology

The RCP manifests as a small, pink, asymptomatic sessile papule measuring <5 mm in diameter, soft in consistency, and without any surface alterations, located on the lingual mandibular cuspid gingiva, and is typically identified incidentally during routine periodontal examinations [2]. Its distribution may be unilateral or bilateral, with variations documented across different ethnic groups [1]. In three Latin American pediatric cohorts, the RCP was observed in approximately 25% of children aged <5 years, with bilateral distribution being more prevalent in the Nicaraguan cohort [1]. Among adults, the prevalence is significantly lower; a study involving 232 adults aged 20-63 years reported RCPs in 9.1% of the participants, with 57% exhibiting unilateral and 43% bilateral distributions [1]. Across the literature, the reported prevalence in children and young adults ranges from 25% to 99%, decreasing to 6-19% in older adults, indicating natural involution with age [1]. A female predilection has been noted across multiple cohorts, although this has not been universally observed [2]. When clinicians can confidently identify the RCP based on its specific location and morphology, no biopsy, treatment, or follow-up is necessary [1,4].

Controversies in histopathology and classification

The primary controversy surrounding the RCP pertains to its microscopic characteristics: histomorphological examination of 30 excised RCPs revealed that approximately 80% contained loosely organized fibrous connective tissue with stellate and multinucleated giant fibroblasts, a pattern indistinguishable from GCF [1,5]. Immunohistochemical analysis indicates that these giant fibroblasts in both entities are vimentin-positive and consistently negative for CD68, S-100 protein, and smooth muscle actin, confirming a fibroblastic lineage and excluding macrophage, melanocytic, or myofibroblastic origins [2]. Given that histopathology alone cannot differentiate RCPs from GCFs, clinicopathological correlation is essential [2]. The critical distinguishing factor remains the anatomical location: GCF exhibits no site predilection and may occur on the tongue, buccal mucosa, or palate, whereas the RCP is invariably confined to the lingual mandibular cuspid attached gingiva [2,4]. The mechanism underlying giant fibroblast formation remains unresolved: evidence from GCF studies, demonstrating proliferating cell nuclear antigen positivity with Ki-67 negativity, supports cell-to-cell fusion over mitosis without cytokinesis; however, whether this mechanism applies equally to RCP giant cells has not been confirmed [5]. Further complicating the issue, given the histological overlap, some archived GCF diagnoses from the mandibular lingual region may represent misclassified RCPs, potentially inflating published GCF prevalence figures [1].

Conclusion

The RCP is a developmental gingival variant that is recognizable on clinical grounds because of its exclusive location in the lingual mandibular cuspid gingiva, asymptomatic nature, small size, and predominance in children and young adults. Treatment is not warranted when a diagnosis is made with confidence. Biopsy is indicated for atypical or doubtful cases. The shared morphology of giant fibroblasts with GCF, unresolved mechanisms of fibroblast multinucleation, and contested nosological status represent genuine knowledge gaps. For dentists, familiarity with the clinical signature of RCPs is the most effective safeguard against unnecessary biopsies and misclassification.
